# Intelligent anti‐epidemic mask based on KF and ECF fusion algorithm

**DOI:** 10.1049/ell2.12240

**Published:** 2021-05-25

**Authors:** Kun Xia, Xiang Li, Xueyong Li, Yiren Liu, Hongwei Zhang, Ruifeng Hou

**Affiliations:** ^1^ Department of Electrical Engineering University of Shanghai for Science and Technology Shanghai People's Republic of China

## Abstract

In response to environmental pollution and the spread of Coronavirus Disease 2019 (COVID‐19), this paper proposes a new type of smart mask design, and specifically proposes an optimized double closed‐loop control method, especially an improved filtering fusion algorithm. Using the filtering fusion algorithm proposed in this paper, after the Kalman filter (KF) filters the raw data of the attitude sensor, explicit complementary filtering and data fusion are used to obtain the attitude angle of the body. At the same time, the obtained attitude angle is combined with acceleration and blood oxygen concentration to obtain the behaviour characteristic value. On this basis, the speed of the oxygen supply fan captured by the photoelectric sensor is used to form a closed loop with the characteristic value of the behaviour. Finally, the structure of the mask is upgraded and optimized through fluid mechanics simulation, and experiments have verified that the combination of the replaceable filter cloth, the intelligent control system and the ultraviolet disinfection device can effectively protect people's health.

## Introduction

Since 2020, Coronavirus Disease (COVID‐19) has spread all over the world. This novel type of virus has not only severely affected the economic development of countries around the world, but has even claimed the lives of countless people. At present, wearing a mask is one of the most effective ways to block virus invasion or respiratory infections [1]. Therefore, how to effectively prevent and control the epidemic has become an international research hotspot. Kalavakonda et al. [2] have proposed a smart mask that eliminates pathogen aerosols near the mouth and nose by actively spraying mist. Kim et al. [3] proposed a smart mask that uses sensors to obtain the temperature and tension of the face to monitor the health of healthcare personnel in real time. Although the above methods have upgraded and optimized the functions of traditional masks, the performance improvement is relatively limited. At the same time, none of the above‐mentioned systems has a mask air volume control strategy and method that matches the perception of human posture movement. Moreover, they also cannot meet the requirements of virus blocking, health monitoring, remote control, and active provision of fresh air at the same time.

**Fig. 1:**
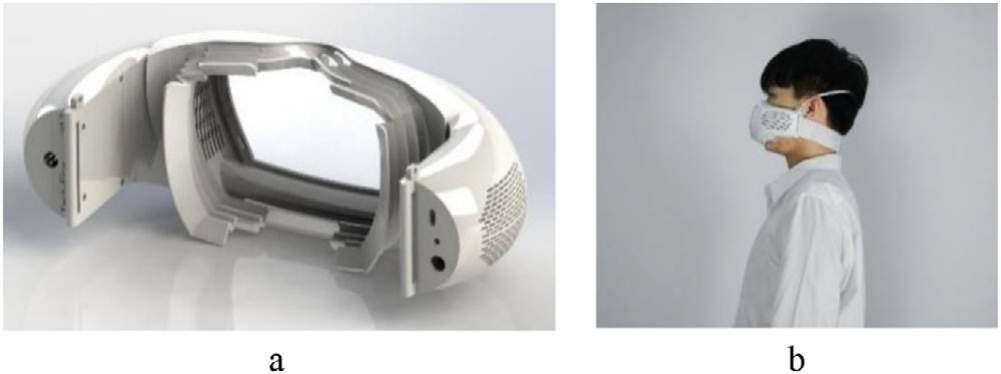
Prototype of a smart mask: (a) Entire structure of prototype, (b) wearing the prototype with ease

In order to solve the above problems, this paper proposes a double closed‐loop control method that adjusts the gas exchange efficiency inside the mask through acceleration, attitude angle and blood oxygen concentration. In addition, the entire control system is installed in a new structure based on fluid mechanics simulation design, as shown in Figure 1a. With the advantages of dual closed‐loop control and ease of wearing, as shown in Figure 1b, the device not only effectively solves the problems of breathing difficulties caused by long‐term wearing, but also provides a way for wearers to monitor their health. The innovation of the smart mask proposed in this paper is the optimized double closed‐loop control method, the novel structure design and the application of the internet of things. This work is of great significance to popularize classic control methods and filtering algorithms in system design and application. In addition, it also has a positive impact on the protection of human health under environmental pollution and epidemic situations.

## Method analysis

In order to ensure that a stable and accurate attitude angle can be obtained under any circumstances, the implementation method of this paper is to filter the original data through the KF algorithm first [4, 5, 6, 7], and then perform data fusion through the explicit complementary filter (ECF) algorithm. This method can not only avoid errors caused by using accelerometers and gyroscopes, but also obtain accurate acceleration and attitude angle information. The process of Kalman filtering could be written as [8]:

(1) $${\hat{x}}_{k}^{-}=A\cdot f\left({\hat{x}}_{k-1}\right)$$

(2) $$P_{k}^{-}=A\cdot P_{k-1}\cdot A^{T}+Q$$

(3) $$K=P_{K}^{-}\cdot H^{T}{\left(H\cdot P_{K}^{-}\cdot H^{T}+R\right)}^{-1}$$

(4) $${\hat{x}}_{k-1}={\hat{x}}_{k}^{-}+K\left(y_{k}-H\cdot {\hat{x}}_{k}^{-}\right)$$

(5) $$P_{k}=\left(I-K\cdot H\right)\cdot P_{k}^{-}$$

where ${\hat{x}}_{k}^{-}$ is the predicted value, $P_{k}^{-}$ is the filter deviation matrix, *A* is the state transition matrix, and *H*is the transition matrix. Additionally, y_*k*_ is the observed value and *I* is an identity matrix. Therefore, *K*that is Kalman gain can be obtained from (1) and (2). *Q* is the covariance matrix of the system noise. This value should be set relatively small so that the six‐axis data of the incoming sensor converges quickly. Since the variables passing through the KF are the components of acceleration and angular velocity on the *x*, *y*, and *z* axes, this paper studies six groups of one‐dimensional state spaces [9]. Therefore, the values of *A* and *H*, and *P*_0_ that is the initial value of *P_k_* are all first‐order identity matrices. Similarly, after a lot of debugging, the value of the first‐order matrix *Q* is 0.01 to meet the design and experimental requirements.

Before performing ECF, the information output by the accelerometer and gyroscope needs to be converted into a quaternion vector. In the process of wearing a smart mask, the process of calculating posture could be written as

(6) $$e=\hat{a}\times \hat{\nu }$$

(7) $$\delta =k_{P}\cdot e+k_{I}\cdot \int e$$

(8) $$\overset{\cdot }{\hat{q}}=\frac{1}{2}\hat{q}⊗P\left(\Omega +\delta \right)$$

where *e* is the error vector. After δis obtained by the proportional integral (PI) operation, it is fused with the output Ω of the gyroscope through explicit complementary filtering to obtain ${\hat{q}}^{\cdot }$, as given in (7) and (8). Finally, the attitude angle of the body is calculated by ${\hat{q}}^{\cdot }$. After a lot of debugging, it is determined that the value of *k_P_* is 10 and the value of $k_{I}$ is 0.00008.

## System design

**Fig. 2:**
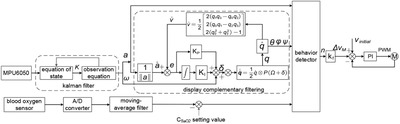
Block diagram of the entire control system

The control system proposed in this paper can detect changes in blood oxygen concentration, attitude angle and acceleration to ensure the real‐time and stable operation of the system, as shown in Figure 2. First, the MPU6050 is used to collect raw data such as acceleration and angular velocity in real time. Then they are sequentially fused with Kalman filtering and explicit complementary filtering. Finally, accurate acceleration and attitude angle are obtained. In addition, the PI regulator is added to the explicit complementary filtering algorithm to form a closed‐loop structure, so that the system can respond quickly to changes of attitude angle. At the same time, in order to improve the accuracy of the output results, a blood oxygen concentration sensor is used to monitor human health in real time, and an alarm will be triggered when the monitoring value is lower than 95%. Further, after passing the processed acceleration, attitude angle and blood oxygen concentration data through the behaviour detector, the real‐time behaviour characteristic value that can be written as *n* can be obtained. Finally, *n* is converted into a compensation value for the speed of the oxygen supply fan through $K_{f}$. Then the compensation value is processed by the PI algorithm together with the initial speed and feedback value of the oxygen supply fan to form a closed‐loop control, thereby realizing the rapid response of the system. In addition, the behaviour characteristic value is determined by different exercise intensities. In the control system proposed in this paper, the value of $K_{f}$ is 1100. $V_{initial}$ means the initial speed of the oxygen supply fan, and its value is 2000. $\Delta V_{M}$means the compensation value of the speed of the oxygen supply fan. The relationship between $\Delta V_{M}$and *n*can be written as:

(9) $$\Delta V_{M}=K_{f}\cdot n$$

## Prototype and experimental results

**Fig. 3:**
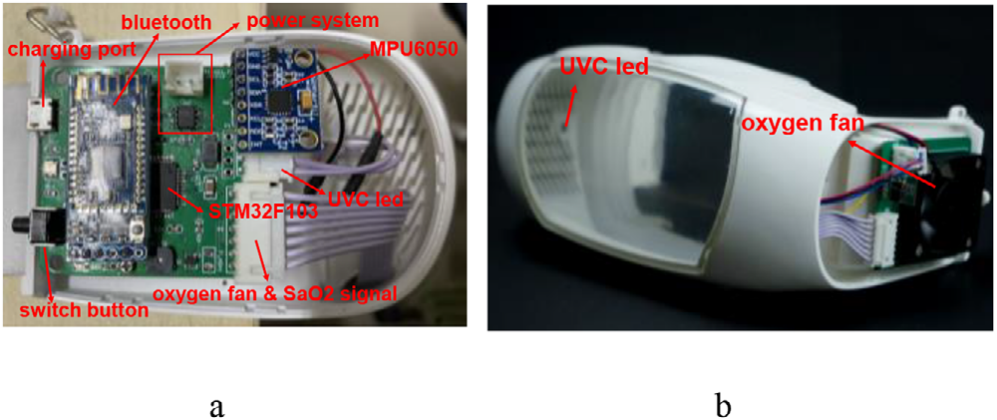
The internal structure of the smart mask: (a) Control PCB of prototype, (b) oxygen fan of prototype

**Fig. 4:**
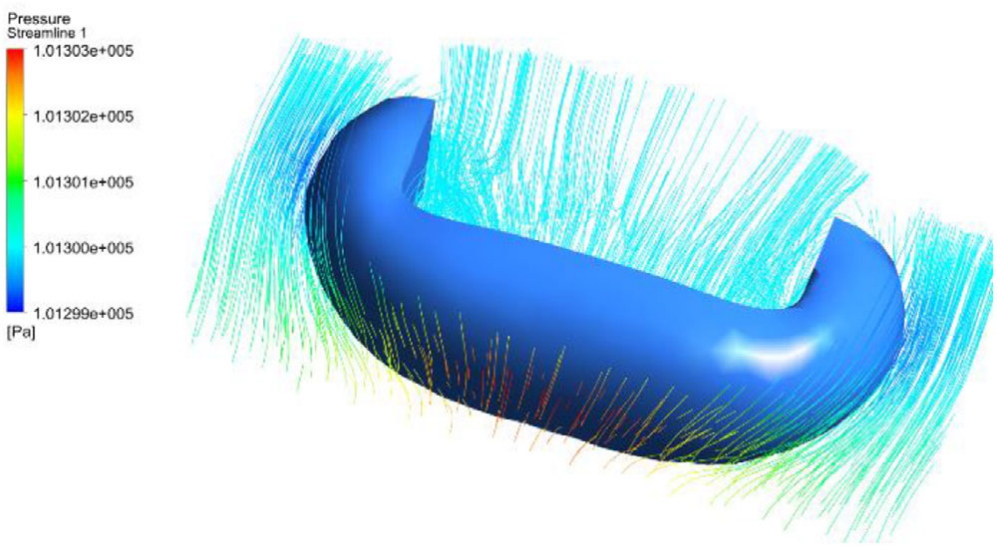
Fluid mechanics simulation results of prototype

**Fig. 5:**
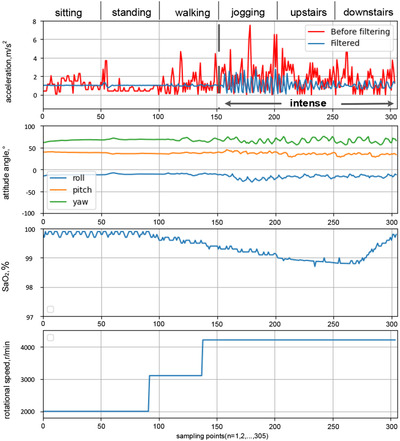
Experiments of the smart mask system in working mode

The control system of the smart mask includes a control printed circuit board (PCB), a monitoring sensor, an oxygen supply fan, and so on, as shown in Figure 3. The control PCB is the core of the entire system and is used to control sensors and the oxygen fan. According to the fluid mechanics simulation results, as shown in Figure 4, the air pressure on both sides of the structure is the lowest. Therefore, the vents are symmetrically arranged on both sides of the structure, and a filter cloth is installed, which can facilitate the exchange of gas inside and outside the mask. In order to be able to adaptively adjust the speed of the oxygen supply fan and improve the compensation efficiency of the air inside the mask, this paper converts the processed value of acceleration, three‐axis attitude angle and blood oxygen concentration into behavioural characteristic values. Then, the speed of the oxygen supply fan is closed‐loop controlled by the behaviour characteristic value. Through 200 dynamic experiments, the functional stability and reliability of the new smart mask system were verified. When the mask is in a different state of exercise, the speed of the oxygen supply fan will increase with changes in acceleration, attitude angle and blood oxygen concentration, as shown in Figure 5. In order to evaluate the prediction performance, this paper employs a commonly used metric: root mean square error (RMSE), as shown in Table 1. It can be seen from Table 1 that compared with the acceleration before filtering, the method proposed in this paper can significantly improve the performance, which shows that this method is effective. This ensures and meets the user's demand for air during exercise. Finally, the state of the system proposed in this paper can be transmitted to the mobile terminal to view through the Bluetooth module, so that users can monitor their own health status in real time. Experiments have proved that the filter fusion algorithm and control method proposed in this paper are superior to other classic filter algorithms and control methods in the motion detection and health monitoring of smart masks.

**Table 1:** Comparison of RMSE of acceleration before and after filtering

Action	Before filtering	Filtered
Sitting	0.61	0.02
Standing	0.77	0.08
Walking	1.14	0.08
Jogging	2.10	0.79
Upstairs	1.30	0.37
Downstairs	1.29	0.25

## Conclusion

This paper introduces a new type of smart mask that can be used in the current environmental pollution situation or epidemic situation. The device changes the gas exchange path of the traditional mask, and further improves the protection efficiency and the reuse rate of the filter cloth. In terms of hardware, the device not only has good battery life, but also can automatically disinfect with ultraviolet light when charging, which makes the mask more intelligent. The double closed‐loop control system proposed in this paper ensures that the system can adaptively adjust the air intake under different behaviours, thereby improving the gas exchange efficiency inside the mask. Experiments show that the device not only has good robustness, but also can realize the function of health monitoring, which will bring convenience to human life during the global epidemic.
